# Iterative Blind Deconvolution Algorithm for Deblurring a Single PSP/TSP Image of Rotating Surfaces

**DOI:** 10.3390/s18093075

**Published:** 2018-09-13

**Authors:** Anshuman Pandey, James W. Gregory

**Affiliations:** Aerospace Research Center, The Ohio State University, 2300 West Case Road, Columbus, OH 43235, USA; pandey.46@osu.edu

**Keywords:** pressure-sensitive paint (PSP), temperature-sensitive paint (TSP), polymer/ceramic (PC-PSP), deblurring, rotating surface pressure measurement

## Abstract

Imaging of pressure-sensitive paint (PSP) for pressure measurement on moving surfaces is problematic due to the movement of the object within the finite exposure time of the imager, resulting in the blurring of the blade edges. The blurring problem is particularly challenging when high-sensitivity PSP with a long lifetime is used, where the long luminescence time constant of exponential light decay following a burst of excitation light energy results in blurred images. One method to ameliorate this effect is image deconvolution using a point spread function (PSF) based on an estimation of the luminescent time constant. Prior implementations of image deconvolution for PSP deblurring have relied upon a spatially invariant time constant in order to reduce computational time. However, the use of an assumed value of time constant leads to errors in the point spread function, particularly when strong pressure gradients (which cause strong spatial gradients in the decay time constant) are involved. This work introduces an iterative method of image deconvolution, where a spatially variant PSF is used. The point-by-point PSF values are found in an iterative manner, since the time constant depends on the local pressure value, which can only be found from the reduced PSP data. The scheme estimates a super-resolved spatially varying blur kernel with sub-pixel resolution without filtering the blurred image, and then restores the image using classical iterative regularization tools. A kernel-free forward model has been used to generate test images with known pressure surface maps and a varying amount of noise to evaluate the applicability of this scheme in different experimental conditions. A spinning disk setup with a grazing nitrogen jet for producing strong pressure gradients has also been used to evaluate the scheme on a real-world problem. Results including the convergence history and the effect of a regularization-iteration count are shown, along with a comparison with the previous PSP deblurring method.

## 1. Introduction

Pressure-sensitive paint (PSP) is a non-contact, optical diagnostic for acquiring time-resolved surface pressure distribution [1,2,3]. This sensor technology is based on capturing the quenching phenomena of the excited state luminescence of oxygen-sensitive luminophores using a high-resolution camera. PSP testing is particularly suitable for pressure measurement on rotorcraft blades where conventional techniques such as pressure taps are difficult to implement and limit the spatial resolution of valuable pressure information [4,5]. When rotating at high speeds, these rotorcraft blades can experience highly unsteady transonic flows with shock waves of varying strength and positions. High-resolution pressure information afforded by PSP testing can be used to identify the location of such sharp pressure differentials with high accuracy. However, the movement of blades during the PSP data acquisition leads to erroneous measurements due to the blurring of the captured intensity image [4,5]. To avoid errors induced by blurring in such situations, mirror-based derotation [6] and deconvolution-based deblurring methods [7,8] have been recently developed. The focus of this work is on improving upon the deconvolution-based method, which currently assumes a uniform blur at all points on the image. This assumption is not appropriate when spatial variation in blur is present, which will be the case when strong pressure gradients such as shock waves are present on the rotating surface. To accurately resolve pressure information in such cases, an iterative blind deconvolution method based on a spatially varying point spread function has been developed and is described in this work.

**Figure 1:**
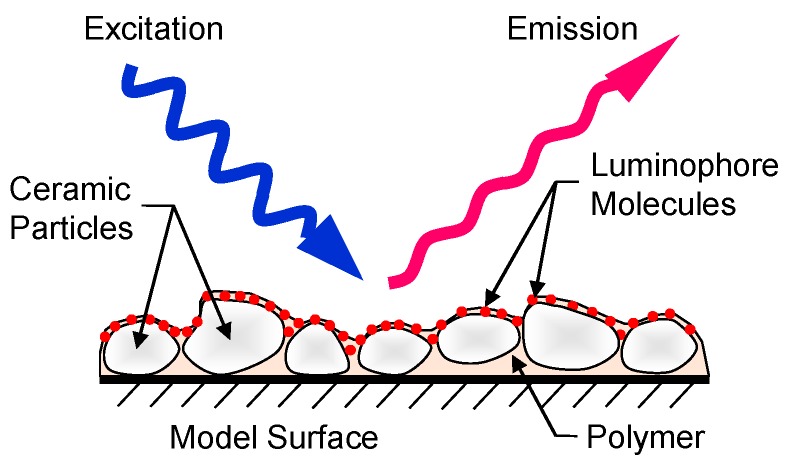
Schematic of basic pressure-sensitive paint (PSP) (adapted from Gregory et al. [3]).

PSP is comprised of luminophores supported on a suitable binder, which is first applied on a blade and excited with a light source; then, the resulting luminescent emission is recorded using an image-sensor such as a charge-coupled device (CCD) camera (Figure 1). The intensity of excited-state luminescence is modulated based on the concentration of oxygen in the vicinity of the luminophores, and this dependence of intensity on pressure is then exploited to quantify the pressure distribution. Thus, every pixel on the CCD camera acts as a pressure probe by storing the luminescent intensity information from a finite region on the blade, which is then converted to a pressure distribution data through the Stern–Volmer equation:

(1) $$\frac{I_{\mathrm{ref}}}{I}=A+B\;\left(\frac{P}{P_{\mathrm{ref}}}\right).$$

here, $I_{\mathrm{ref}}$ and $P_{\mathrm{ref}}$ are the reference values of intensity and pressure, respectively, taken at a “wind off” reference condition, in order to counter the spatial intensity variations due to non-uniform paint thickness or illumination, which cancel out in the intensity ratio. The calibration coefficients *A* and *B* are typically functions of temperature. Improvements in the oxygen permeability of binders has led to the development of fast-responding versions of PSP (Fast-PSP), which enable measurements of unsteady pressure fields [3]. Polymer–ceramic pressure-sensitive paint (PC-PSP) is a commonly used Fast-PSP with response times as low as 100 µs [9,10], and has been used to study unsteady aerodynamic phenomena acting on rotating surfaces [4,7,11]. To enable this application, a single-shot lifetime method based on pulsed laser excitation was developed in order to counter inherent problems such as the shot-to-shot variation of laser illumination, the cycle-to-cycle variation of wind-on positions, and the qualification of unsteady content not periodic with rotation frequency. This single-shot method employs a charge-coupled device (CCD) camera with a short first exposure of controlled duration, for referencing the subsequent long open-ended second pressure-sensitive gate. For resolving helicopter aerodynamic problems of interest using this self-referencing lifetime-based method, platinum porphyrin (PtTFPP) in polymer/ceramic has been used for the PSP [9], which has good sensitivity at the cost of a longer lifetime of luminescent emission. Temperature-sensitive paint (TSP) operates using the same principle of luminescence quenching, but due to thermal effects, has also been used on rotor blades in single-shot mode for temperature measurements [11,12].

When the single-shot implementation of PSP/TSP is used on high-speed rotating surfaces, image blurring is inevitable. A pixel of the CCD imager that would capture emission from a single finite region on a stationary blade ends up accruing luminescence intensity from several finite regions on the surface as the blade moves. In several studies, longer lifetime PSP is used [7,11,12] to improve upon the sensitivity of the pressure measurements, which further exacerbates the blurring problem. Blurring also arises in continuous illumination PSP measurements [13] where longer exposure times are usually used to improve the signal-to-noise ratio (SNR). For an ideal case—where the rotating blade has uniform illumination, uniform paint thickness, and uniform pressure throughout—every point on the blade would emit an equal number of photons at any instant. In this degenerate case, the blurred intensity captured by an Eulerian probe such as a CCD camera pixel can be accepted as the Lagrangian intensity emitted by the corresponding region. However, this does not hold in the real-world case of spatially varying pressure fields, because the Eulerian and Lagrangian measurements would be different. This is particularly not true for regions near the edges of the rotating surfaces. Since pressure information is extracted by the amount of luminescent intensity captured by each pixel in the recorded image, it is critical that the correct intensity value be restored through spatially resolved deblurring techniques.

Three techniques have recently been developed in order to counter the blurring issue: (1) short-exposure double-framing from modified interline-transfer CCD cameras [14,15], (2) mirror-based derotation [6], and (3) deconvolution-based deblurring [7,8]. In the field of camera architecture, Geisler [14] and Weiss et al. [15] developed a firmware modification for interline-transfer CCD cameras that enables the independent determination of the timing events for the two successive image frames. Thus, the requirement of an open-ended second image of the interline camera is obviated, and the user has direct control of the exposure of both frames. This is a very effective approach for controlling the image blur for single-shot lifetime PSP measurements. However, this development has not yet proliferated to all of the commercially available camera architectures. Furthermore, there remain many measurement situations when a longer exposure is desirable (or even required), but with a concomitant susceptibility to image blurring that must be addressed.

Mirror-based blur prevention techniques are physical methods that employ a moving mirror that ensures that each pixel on a fixed camera sees the same finite region on the moving surface throughout the exposure period. For a particular blur type, mirror movement needs to be predetermined, and a mirror that can follow the required trajectory during the exposure period needs to be designed before the wind tunnel measurements can be performed. For rotor measurements, the mirror can be either co-aligned with the rotor axis (on-axis), or it can be off-axis with the axes of rotation of the mirror and rotor coinciding at the rotor hub. A detailed comparison study was performed by Pandey et al. [16] to compare the mirror-based derotation and deconvolution-based deblurring techniques for single-shot TSP measurements. It was found that while the on-axis configuration of the mirror is easier to use, it increases the distance between the camera and the rotor blade, which reduces the PSP luminescence signal levels captured by the camera. On the other hand, the off-axis configuration requires the use of a selective trigger (since the rotor rotational rate is not an integer multiple of the mirror rotational rate in this configuration) or a galvanic mirror (which is usually expensive and does not move at the appropriate speeds required in rotorcraft studies). However, as demonstrated first by Raffel and Heineck [6], it was found that if an appropriate mirror can be obtained and aligned, derotation is very effective at preventing the blur in rotor measurements, and when the luminescent signal levels are low, it is a much better option than the post-processing blur removal [16]. This technique has subsequently been used in continuous light TSP [17] and infrared measurements [18].

Deconvolution-based deblurring methods, on the other hand, are post-processing methods that seek to minimize the blur in the captured images. Since no camera–mirror alignment is required, these methods are economical and save wind-tunnel testing time. These are also applicable at different speeds and movement types, where a particular type of mirror might fail. In the comparative study of Pandey et al. [16], it was shown that when working with good luminescent signal levels, both derotation and deblurring methods are equally effective at blur elimination. This technique has been used in single-shot PSP [7,8,11,12] and TSP measurements [12,16]. The focus of this work is on improving upon the deconvolution-based method for application with sharp pressure gradients such as the identification of the location of a shock on a rotor blade. The deblurring method currently being used assumes a uniform invariant blur at all of the points on the image. However, in actuality, the blur at every pixel dependents on the local pressure, which is spatially varying, and the invariant deblurring method fails when sharp pressure differentials are present [8]. Moreover, the assumed value of point spread function (PSF) might not be appropriate, which would lead to an inaccurate restoration of the blurred image. In the following section, the motivation for the current work is provided by reviewing the technical difficulties of the problem at hand, and the inadequacy of the current state-of-the-art deblurring algorithms at addressing it.

## 2. Image Deblurring and Need for Current Work

Image deblurring belongs to an important class of ill-posed linear inverse problems that take up the form of Fredholm integral equations of the first kind [19]. The discretized general blurring model of the forward problem is represented as:

(2) L=K∗S+n

where *S* is the sharp image, *K* is the blurring kernel composed of the point spread functions (PSFs) that convolve (*) with *S* to produce the blurred image *L*, and *n* is the additive noise during this imaging process. Image deblurring is the inverse problem of obtaining *S* from *L*. A major issue leading to the ill-posedness of such problems is stability: the singular values of *K* in image deblurring problems tend to decay to zero and amplify the high-frequency noise, which corrupts the restored image with significant noise [20]. If the kernel is invariant and favorably structured, and if the blurred image is noise-free, fast Fourier transform (FFT)-based fast deconvolution can be employed [21]. However, more realistic (noisy) inverse problems are very sensitive to noise amplification, and require the appropriate use of regularization techniques such as truncated singular value decomposition (SVD), Tikhonov, or Weiner methods to spectrally filter noise-dominant frequencies [20]. For larger images, since it is computationally infeasible to obtain the SVD of the associated K matrix, either a variational form of regularization or iterative methods [22,23,24] are used. In the present work, iterative tools developed by Nagy and coworkers [25,26] have been used.

Apart from the challenges of noise and image size, the complexity of the image deblurring problem also depends on the knowledge and structure of the blurring kernel. The spatially varying image blurring model first formalized by Lohmann and Paris [27] represents a more general problem in which the PSFs in *K* are *not* invariant, but depend on the location of a pixel in *S*. Previously, the problem has been made tractable through the use of a coordinate transformation to make the blur invariant [8,28,29], or by using the invariant restoration of sections of the image, which are subsequently stitched together [30]. A similar approach of invariant sectioning was introduced with PSFs instead for a smoother restoration [31], and the resulting problem was solved using the preconditioned conjugate gradient method (CGLS). Blind deconvolution problems arise when the information about the PSFs, and hence the blurring kernel *K*, are not known completely. In this severely ill-posed problem, both *S* and *K* in Equation (2) have to be found out from only *L* and an estimate of noise. In the field of blind deconvolution, many state-of-the-art algorithms assume invariant blurring [32,33] to simplify the problem; however, as expected, this assumption is often violated [34].

Spatially varying blind deconvolution problems have relied on measures to increase the amount of available information through either multichannel methods, which use multiple images of the same sharp scene but blurred differently [35,36], or through supplemental sensors to help define the blurring kernel [37]. Single image approaches [38,39,40] try to recover a variant blur kernel based on the detection of edges and prediction of the underlying sharp edges. Shan et al. [40] found the rotational blur kernel for a single image by separating the foreground (rotating object) from the background (fixed plane), which was then used to define a transparency map-based motion descriptor. However, their assumption that the transparency map between the foreground and background could be used to estimate the blur will fail for PSP deblurring with pressure gradients. In spatially varying pressure fields, the PSF estimated from the edge-based estimator will not be sufficient for deblurring PSP luminescence in the interior of a blade surface, since it would be different from that at the edges. As pointed out by Sroubek and Milanfar [41], the common approach of state-of-the-art single-image deblurring methods is to predict strong edge; however, the absence of salient edges or corruption by noise leads to their failure.

The application of PSP on rotating surfaces leads to blurring that can be depicted accurately only through a spatially varying blur kernel. The blurring kernel is comprised of PSFs that depend not only on the frequency of rotation and second gate exposure of the CCD, but also on the local emission lifetime. Although the first two are known for a wind tunnel run, the time scan of intensity emitted by a point is inherently unknown, since it depends on the pressure experienced by the point during the receptive second gate. The exponential decay of intensity undergoing rotational blur also makes PSP blurring more complex than a simple solid-body rotational blur. A first-order technique was used by Juliano et al. [7] and Disotell et al. [11], where a radially varying and column-wise constant PSF was assumed, and used for deconvolving the blurred PSP image. Coordinate transformation was introduced by Gregory et al. [8] to convert the blur to only one coordinate, and an assumed invariant kernel was then used to show effective results for the images of small pressure differentials. However, both of these deblurring approaches eschew the estimation of the blur kernel by using an assumed value of pressure (which itself is to be measured) to determine the PSFs at each point. In order to reap the benefits of a self-referencing lifetime-based method, effective methods to remove the rotational blur of spatially varying exponentially-decaying intensities need to be developed. Variation in illumination, paint thickness, and surface pressure over the rotating surface cause variations in the local lifetimes of emission, which makes it necessary that pixel-to-pixel spatially varying blind deconvolution methods be used for the effective deblurring of PSP images. A review of image deblurring methods reveals that there is no spatially-varying blind deconvolution technique, that can be effectively employed on only a single (second-gate) PSP image, to accurately resolve the surface pressure maps on rotating surfaces. Motivated by this necessity, the present work was undertaken to understand the mechanism of blurring in single-shot PSP images on rotating surfaces, and develop a reliable deblurring scheme. In this work, an iterative scheme has been developed that converges, with great accuracy, to the sharp pressure profile while using regularization to curb the effect of noise in the imaging process.

## 3. Approach to Iterative Deblurring

The iterative scheme is based on the lifetime characteristics of PSP, which form the basis of the single-shot measurement method. So, in the first subsection, the workings of the single-shot method and PSP calibration are explained. The subsequent subsection provides detail about blurring kernels, and then explains the procedure to generate one when the correct point spread functions of the image are known. The concluding subsection provides details of the iterative scheme—i.e., the procedure of iteratively obtaining the point spread functions along with the regularization tools used in this work for the suppression of noise from corrupting the deblurring process.

### 3.1. Single-Shot Method and PSP Characteristics

**Figure 2:**
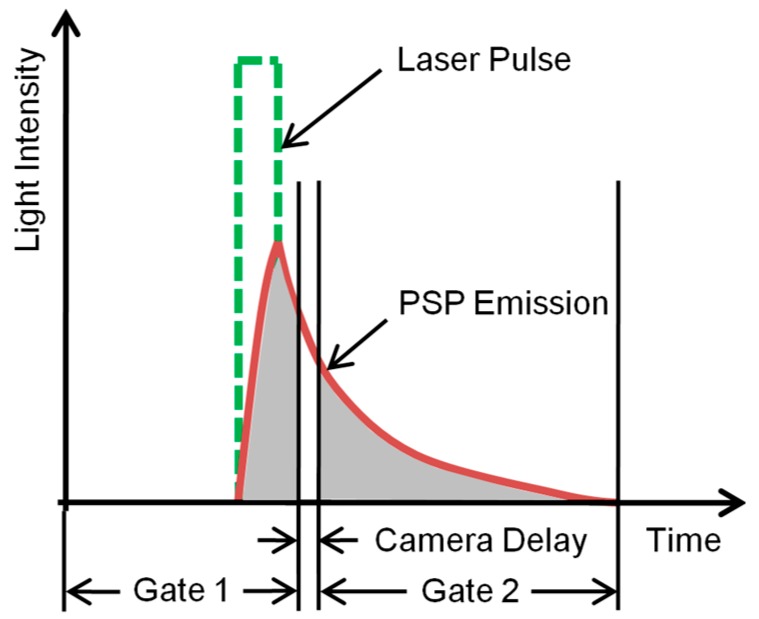
Single-shot lifetime PSP method (adapted from Juliano et al. [7]).

The single-shot method is based on the self-referencing of PSP images that are recorded after a single shot of high-intensity laser illumination. The technique (Figure 2) comprises two exposures: $G_{1}$ represents the intensity captured during Gate 1 of a CCD camera; it integrates the initial pressure-insensitive light emission from the PSP ($I_{\mathrm{Gate}1}$) and serves as reference for the long, open-ended pressure-sensitive Gate 2 ($G_{2}$), which picks up the photons emitted during the lifetime decay of luminophores from their excited state (represented as $I_{\mathrm{Gate}2}$). CCD cameras have an inherent time delay between the two gates, which leads to a loss in captured intensity ($I_{delay}$). The exposure duration of Gate 1 may be adjusted on a double-framed camera to balance the light intensity in both gates at ambient conditions, capturing maximum pressure sensitivity while minimizing the effects of imager shot noise. A wind-off reference ratio with same imager setting is mapped to the wind-on ratio to further eliminate the spatial variation with the resulting ratio-of-ratios used in the modified Stern–Volmer equation:

(3) $$\frac{{\left(I_{\mathrm{Gate}2}/I_{\mathrm{Gate}1}\right)}_{\mathrm{ref}}}{\left(I_{\mathrm{Gate}2}/I_{\mathrm{Gate}1}\right)}=\frac{{\left(G_{2}/G_{1}\right)}_{\mathrm{ref}}}{\left(G_{2}/G_{1}\right)}=A\left(T\right)+B\left(T\right)\frac{P}{P_{\mathrm{ref}}}$$

**Figure 3:**
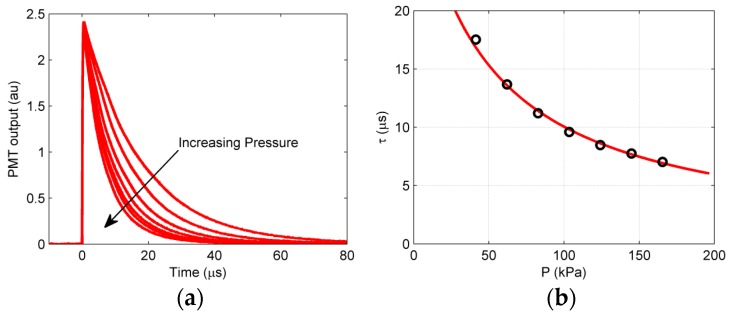
Measured lifetime decay curves (**a**) and corresponding lifetime calibration values (**b**) for polymer-ceramic (PC)-PSP with PtTFPP (adapted from Gregory et al. [8]).

For a detailed procedural flowchart for data acquisition and post-processing of the single-shot method, refer to Juliano et al. [7]. A complete knowledge of the PSP calibration is a critical input to not only the iterative scheme, but also for the conversion of intensity to pressure data. These characteristics include pressure and temperature sensitivities along with the lifetime constant as a function of pressure. Figure 3 shows the luminescent lifetime variation (with pressure) for the PSP used in this work (PtTFPP on polymer/ceramic). For details on the calibration process and other calibration characteristics of this PSP formulation, the reader is referred to Gregory et al. [8]. It can be noted that as the pressure increases, the higher partial pressure of oxygen increases the probability of quenching of luminophores, thereby reducing the apparent emission time scale of PSP. This modulation of local PSP lifetime by local surface pressure manifests as a variation in PSFs across a blurred single-shot image. Since the decay lifetime of PtTFPP PSP is usually shorter than the temporal azimuthal pressure differential over a rotating surface, it can be safely assumed that a point experiences a constant pressure throughout its lifetime, and the spatial variance in decay rate arises only due to the different surface pressure experienced by different points. A sharp change in pressure such as due to a presence of a shock wave can cause large changes in local lifetimes (and thus PSFs) across the shock. Based on the lifetime characteristics (Figure 3), one can expect that blurring would increase if the pressure is lower; this relationship between lifetime and PSF is detailed in the next section.

### 3.2. Spatially Varying Kernel

A general blurring model takes the generic form [20]:

(4) $$l(x)=\int_{0}^{1}\int_{0}^{1}K(x,y)s(y)dy_{1}dy_{2},y\in \left[0,1\right]\times \left[0,1\right]$$

where *x* and *y* are coordinates of the blurred and sharp images, respectively (assuming the domain to be [0, 1]). This linear relationship between a blurred image *l* and its latent image *s*, through the blurring kernel *K*, allows discretization and representation in matrix terms. The resulting kernel-based forward model (Equation (2)) provides an approach to model the blurring process by positioning appropriate PSFs at accurate locations in the kernel. In complex motion blur schemes such as rotational motion with varying intensity, a kernel-based model is difficult to implement due to a need for a sub-pixel working regime to accurately model the contribution of each pixel in the sharp image to the overall blurriness [27,42]. However, the effective transformation of coordinates [8,29] and change of orientation of the blurred PSP image for converting the circumferential blur to one-dimensional poses an easier initial working problem.

**Figure 4:**
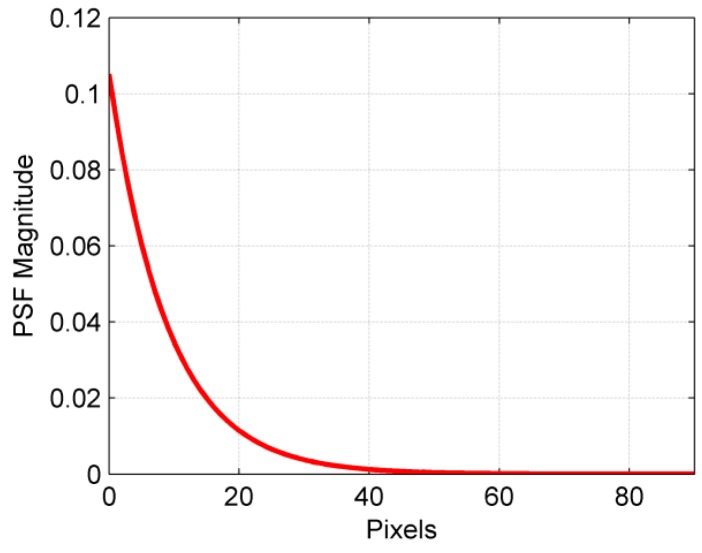
Normalized point spread function (PSF) at atmospheric pressure with a rotational frequency *f* = 269 Hz and length of 10 lifetimes (adapted from Gregory et al. [8]).

For transforming a PSP image to polar coordinates, the part of the image that completely circumscribes the rotating surface is selected such that there is sufficient information to recover the potential (degraded) information. The polar lattice used has a sufficiently higher resolution to ensure the sub-pixel working regime, and follows a *θ*-*r* convention that ensures column-wise blur instead of row-wise. Resolution depends on the computational ability; however, high resolution in the theta direction was ensured to capture the blur accurately. Following Equation (2), the column-wise lexicographically stacked vector-form of this transformed image is denoted by *L*. Each pixel of *L* is obtained from a weighted sum of the corresponding pixel and its neighbors in the sharp image (*S*), and these weights are given by the elements in the blurring kernel (*K*). The alignment of PSFs in *K* can be conceptualized as described in Hansen et al. [43]:

(5) $$Ke_{i}=K(:,i)=\mathrm{column}\;i\;\mathrm{of}\;K$$

where *e_i_* is the *i*th unit vector consisting of all of the zeros with 1 only at the *i*th location. For our column-wise one-dimensional blur, the *i*th column of *K* contains the PSF of the *i*th pixel that starts from the main diagonal and contains the weights that describe how its intensity affects the pixels below it. If the pressure value at a point is known, as is the case for a non-blind deconvolution problem, the PSF can be constructed using the information about lifetime curves, rotation frequency, sub-pixel resolution in the circumferential direction, and the exposure period [8]. A typical normalized point spread function for atmospheric pressure with a rotational speed of 269 Hz and an angular resolution of 0.1/pixel is shown in Figure 4. The lengths of the PSFs were extended over 10 lifetimes at every pressure beyond which the intensity was assumed to be negligible; the resulting PSF was then normalized. This process was automated with a MATLAB function that readily generates the PSF vector when provided with appropriate inputs for a pixel-to-pixel spatially variant kernel.

Both the reconstruction of an image and the structure of the *K* matrix depend on the type of boundary condition used, which specifies the behavior of the scene outside of the boundaries of the given image. Since safe radial and azimuthal margins have been used in this work before converting the PSP image to polar coordinates, no information would be lost if the zero boundary condition were used, i.e., if the exact image was black (zero intensity) outside the boundary. Upon employing this boundary condition, a lower block triangular *K* matrix with triangular blocks is obtained such that the PSFs corresponding to every column of the image form a block. This can be readily solved by forward substitution for a sharp image if the imaging process is noise-free or when the condition number is low enough. Diagonal elements, which are the first elements of every PSF, become the eigenvalues of the *K* matrix. Since *K* is not a normal matrix, the eigenvalues cannot be used to calculate the condition number; instead, MATLAB functions such as rcond and condest can be used to estimate the ill-posedness of the problem. These functions use an iterative algorithm to estimate the norm of the *K*^−1^ matrix without directly estimating *K*^−1^.

The use of sub-pixel resolution leads to a *K* matrix that is of huge dimension, but only with small support. Such a matrix can be efficiently represented by using the sparse matrix representation in MATLAB, which would have been otherwise infeasible (a *K* matrix for images of 600 × 800 dimension would have 480,000 × 480,000 elements, which is well beyond the maximum real double-array holding capacity of a standard PC). MATLAB uses a compressed-column data structure to store sparse matrices, and thus solves the noise-free problem by accessing the *K* matrix column-wise instead of by forward substitution. Although blurring is said to be worse when the support gets wider as singular values decay faster, even for narrow PSFs with a slow decay in singular values, the condition number becomes large for larger images, requiring the use of regularization tools [43].

### 3.3. Iterative Scheme

The iterative blind deconvolution scheme presented in this paper is based on the monotonic calibration curves of PSP formulations. The iterations require the same four images—wind-on and wind-off Gate 1 and 2 images—that are conventionally used in single-shot PSP experimentation to extract pressure information. However, it should be pointed that the scheme is still a single image blind deconvolution, because only the blurred wind-on Gate 2 image contains the information about the sharp Gate 2 image. This is in contrast to multichannel deblurring algorithms that use several differently blurred images of the same scene [41]. The other three images are also transformed to polar coordinates using the same sub-pixel resolution as that of the blurred image.

The scheme starts by initializing *K* with an invariant atmospheric pressure assumption. PSFs are generated, and the *K* matrix is filled with the zero boundary condition. The polar transformed blurred wind-on Gate 2 image is then deconvolved with *K* to perform a first-order deblurring, as in Gregory et al. [8]. This restored image is processed to extract pressure information in the following steps: it is first median-filtered to suppress the white noise while preserving the edges, and then it is registered over the polar transformed wind-on Gate 1 image to obtain a ratio of intensities, after which this ratio itself is registered on the polar transformed wind-off intensity ratio to extract the pressure information through the Stern–Volmer equation (Equation (3)) and calibration curve (Figure 3). This pressure data at every pixel is then used to create PSFs for the *K* matrix of the next iteration, as discussed in the previous section. The updated *K* matrix is used to deblur the polar wind-on Gate 2 image to generate an improved restored image, which again goes through the same processing steps before the next iteration. The monotonic lifetime calibration curves ensure convergence to the sharp Gate 2 image, and a reasonably fine pressure change at every pixel can be used as a stopping criterion of the iterations.

The fundamental difference between this scheme and other state-of-the-art, single-channel deconvolution methods is that the blurred image is not altered at all. Since valuable pressure information is present in the wind-on Gate 2 image, the use of filters, as done in spatial domain methods [39] to suppress noise and overemphasize the edges, should be avoided. In the present scheme, the same unfiltered wind-on Gate 2 image is restored over all of the iterations, albeit with an improved kernel that is obtained through the processing work on the restored image of the previous iteration. Image registration is carried out through the control point registration of the Image Processing Toolbox in MATLAB. To ensure the automation of the iterative process, the control points are selected and saved during the initialization step; then, the same points are used over subsequent iterations. Image registration is carried out in polar coordinates rather than after transforming our deblurred image to Cartesian, in order to obtain a pressure map that is highly resolved. This sub-pixel resolution of PSFs enables single-image super-resolution by deconvolving upsampled images with the recovered blur kernel [38].

There are significant numbers of pixels in the polar-transformed image that capture the stationary background of the experimental setup due to the safe margins employed before transformation. Since PSP experimentation is carried out in the dark, these pixels have very low intensity. A unit vector with one at the main diagonal can be used as the PSF for these pixels to represent that they do not spread. A circumscribing mask created from the Cartesian Gate 1 image with ample room for rotation of the blade during first exposure can be used for bodies that do not present easier geometry when transformed to polar coordinates. This mask can then be transformed to polar coordinates using the same grid to locate the pixels that correspond to the background. To ensure convergence, upper and lower bounds on the pressure range are enforced such that unreasonable intensities (if present due to ringing at blade edges) are eliminated. Bound limits can be problem-specific, and were set at 50% higher (lower) than the corresponding intensity values for the maximum (minimum) expected pressure values. Thus, there are two categories of neglected pixels: the first category captures the background pixels, and the second category includes all of the pixels with values that are out of bounds. In order to ensure convergence, the kernel must be specifically tailored to handle each category of pixels, which is described as follows.

Filling the *K* matrix for the first category (background pixels) is straightforward, as a sparse diagonal matrix is used that has ones for the columns corresponding to the background pixels and zeros for the rest. For the second category (out-of-bounds pixels), the contribution to the *K* matrix is is formed using a sparse invariant blur matrix which is then post-multiplied with a positioning matrix. The sparse invariant matrix is created with the PSF obtained from the mode of the pressure values in the out-of-bounds category. A code was made to readily fill this invariant matrix while preserving the block triangular form of the zero boundary condition. The positioning matrix is a sparse diagonal matrix comprising ones for the columns that need to be preserved and zeros for the columns that should be eliminated. The positioning matrix for the out-of-bounds pixels is then multiplied to the invariant matrix to clear the columns that do not correspond to these pixels. The resulting sparse matrix is then added to the *K* matrix from the first category to update the columns.

The efficient filling of the remainder of the *K* matrix is done by first creating a triplet of a kernel value vector, a row location vector, and a column location vector, and then calling sparse in MATLAB instead of updating the *K* matrix every time [44]. The kernel value vector comprising stacked PSFs is obtained by concatenating the PSFs as they are calculated from the pressure value of the pixels, while the location vectors ensure that every PSF starts at the main diagonal of the *K* matrix and extends below it. It should be noted that the first category of pixels (background) were pre-filled in order to reduce the computational effort in concatenating PSFs. Due care should be taken that only those pixels that can be confidently ascertained as background be used. Since PSFs are generated from the pressure map and the pressure values that correspond to background pixels are meaningless, these can be discarded if it is feasible to reduce the computational effort without any loss of accuracy. On the other hand, the failure to identify all of the background pixels will only produce trivial error, due to the negligible intensities that get spread with the PSFs corresponding to those pressures.

Once the complete *K* matrix is generated, the transformed blurred Gate 2 image is deblurred using the iterative restoration methods developed by Nagy and coworkers [25,26]. The use of regularization over every step of kernel estimation to suppress noise amplification is fundamentally different from the blind deconvolution methods that neglect noise while estimating the kernel and then apply a classical method for restoration. These algorithms are vulnerable to noise and break down with even moderate levels of noise [41]. SVD-based direct filtering is impossible for large matrices, since even though *K* is sparse, the orthogonal matrices obtained by the SVD are not. Moreover, iterative methods have the advantage of imposing new constraints such as non-negativity, or can be used with preconditioners. The regularization is based on the semi-convergence behavior of iterative methods with respect to relative error when applied to the least squares problem:

(6) $$\underset{s}{\min}{\left\|l-Ks\right\|}_{2}$$

The index for stopping the iterations acts as the regularization parameter by defining the size of the singular values that are to be neglected. Since the SNR in PSP images is subject to huge variations, it can be experimentally determined and set in the initialization step, and then the same value is used in the subsequent iterations to ensure automation. The choice of iterative method was made by its applicability to the blind iterative scheme developed here. Although Krylov subspace-based methods—such as the conjugate gradient method for least squares (CGLS) or the bidiagonalization-based least squares (LSQR)—converge very quickly, they also exhibit a sharp increase in noise amplification after achieving this semi-convergence [42,45]. On the other hand, Richardson iterations being inherently slow are also significantly gradual in reconstructing higher frequency detail, and thus, the amplified noise dominates the reconstructions at a much slower rate. Since this work uses a preset value for the regularization parameter, which might not be accurate over all of the iterations, it is prudent to use a method that does not exhibit sharp amplification of noise after achieving the semi-convergence, especially while using it with a low SNR experiment. Consequently, every iteration of the blind deconvolution scheme employs the steepest descent implementation of the Richardson method. More about this classical iterative method and how it can be interpreted as SVD filtering is described by Berisha and Nagy [25], along with the MATLAB notes on its implementation.

## 4. Methodology for Assessment of the Deblurring Technique

In order to test the deblurring scheme, both numerically generated test images with known pressure fields and experimental images have been used in this work. The first subsection explains the numerical approach; a forward model of blurred image generation along with the technique of simulating the effects of a CCD camera (splitting of intensity due to the gating process and the addition of imaging noise) has been described. The subsequent subsection explains the experimental framework that is used to obtain a blurred image with a sharp pressure gradient for testing the scheme on a real-world problem.

### 4.1. Forward Model

In order to test the blind deconvolution scheme developed in this work, an experimental Gate 2 image would suffice as an input. However, in order to validate the resolution and accuracy with which the pixel-to-pixel intensities are restored, both the blurred image as well as its latent sharp form are needed. Thus, numerically-generated pseudo-pressure images are generated and run through a blurring routine (termed a forward model) in order to generate a known data set of sharp image, blurred image, and blurring parameters. However, “Inverse Crime” is referred to as the mistake of using the same precise model both to generate the test data and compute the reconstructions [20]. Kernel-based deblurring has been used in this work to restore images; thus, blurred images have been generated using a kernel-free forward model. The forward problem of creating a blurred image from a known sharp image also helps with understanding the blurring process in PSP images.

Every pixel in a recorded image is proportional to the number of photons accumulated by the corresponding pixel sensor over the exposure period of the imager. An image formation model can be assumed as a binning of these photons into infinitesimally small time intervals, such that the formed image is an integral of the sub-images projected from the real world onto the two-dimensional plane. A discrete form of this model with a sufficiently high number (*N*) of sub-images (*S*) can safely represent the image (*L*) logged in the camera during its exposure time.

(7) $$L=\sum_{i=1}^{N}S_{i}$$

This image formation model can be used to simulate the blurring process [42] provided that the sub-images that represent the motion of the body during the exposure period can be accurately constructed. This kernel-free image degradation model (Equation (7)) is fundamentally different from kernel-based models (Equation (2)), and is more physically intuitive in complex blurs such as rotation. Blurred PSP images of rotating surfaces can be constructed in a similar way using the discretized locations and intensities of the sub-images over the second exposure. Following Tigkos [42] and Whyte et al. [46], this can be represented as:

(8) $$L(x)=\left(\sum_{i=1}^{N}I_{0}\left(H_{i}x\right)\omega_{i}\right)/\left(\sum_{i=1}^{N}\omega_{i}\right)+n$$

where the summations are done over all of the sub-images (*i* = 1 to *N*) with $H_{i}$ being the homography induced by the planar rotation of a sharp PSP image with intensity $I_{0}$. $\omega_{i}$ are the weights of the summation that model both the time spent at the *i*th sub-location and the intensity value at that sub-location, and *n* is simulated noise. For steady frequency rotations, the time spent at each location is the same, while exponentially varying weights can be used to model the decay of intensity. **x** is the homogeneous vector used to denote points on the sensor (points on the observed blurry image *L*). Since the same numbers of photons are captured during the blurring process as would have been for a sharp image, the sum of the weights is used to normalize the intensity to that of the sharp image $\left(I_{0}\right)$. For the construction of sub-images in this work, bilinear interpolation with a large number of sub-images has been used, which provides fine accuracy in modeling the degradation [42].

The heuristic image model (Equation (8)) has been modified to simulate the splitting of the overall intensity $I_{0}$ of luminescent emission into sharp Gate 1 and Gate 2 images:

(9) $$G_{1}(x)=\left(\sum_{i=1}^{N_{1}}I_{0}\left(H_{i}x\right)\omega_{i}\right)/\left(\sum_{i=1}^{N}\omega_{i}\right)+n_{1}$$

(10) $$G_{2}(x)=\left(\sum_{i=N_{1}+1}^{N}I_{0}\left(H_{i}x\right)\omega_{i}\right)/\left(\sum_{i=1}^{N}\omega_{i}\right)+n_{2}=I_{0}-G_{1}-I_{\mathrm{delay}}$$

where the numerator in Equation (9) and Equation (10) are summed over the first and second exposures, respectively, while the denominator is summed over both the gates and delay. To avoid the usual image registration steps done with PSP images for extracting pressure information, a sharp $G_{2}$ image has not been rotated to a position that the rotating surface would have achieved after rotating over the period of the first exposure and camera delay. The accurate splitting of intensities in Gate 1 and Gate 2 is critical to the working mechanism of PSP, since their ratio is used to extract the pressure information from Equation (3). The camera delay between gates 1 and 2 in modern CCD cameras is of the order of 1 ns. To simulate the loss in captured intensity ($I_{delay}$) due to this delay, a forward model was made using 10^9^ images per second, and then, a single image was deliberately neglected. The intensity split with such a high number of images can be readily obtained from a single pixel of intensity 1; the resulting fractional gate intensities can then be multiplied to the sharp image with intensity $I_{0}$ to obtain the sharp $G_{1}$ and $G_{2}$ images.

The short first exposure of the CCD imager in single-shot lifetime PSP experimentation is set such that the intensity is distributed approximately equally in both images. The lifetime decay of PtTFPP in polymer/ceramic, although longer than other PSP formulations, is still short enough that it loses half its intensity in 6 μs at atmospheric pressure. Hence, Gate 1 PSP images experience negligible blurring, and only the sharp Gate 1 images have been used in this study. However, the blind deblurring scheme developed here can also be used for deblurring Gate 1 images, if required for longer-lifetime PSP formulations. Estimation of the Gate 1 blur kernel, once the Gate 2 blur kernel has been derived, is trivial. The blur kernels will be similar in the sense that the point spread functions depend on the pressure experienced during the exposure, which will be the same for both gates 1 and 2, but their lengths will be different, depending on the exposure period. The two blur kernels can be improved simultaneously in an iteration of the scheme described in the previous section.

The degraded Gate 2 image is obtained from Equation (10), with both the summations running only over the second exposure. Since spatially variant blurs are also linear [27], spatially variant degradation can be simulated by the superposition of separately blurred patches of a single image. This procedure can be used to model complex surface pressure phenomena such as a rotor blade with a shock present. For modeling experimental error, noise may be added to the noise-free blurred image using the imnoise function in MATLAB’s Image Processing Toolbox or the built-in randn function. A commonly used additive noise model [43] for CCD arrays includes (1) Poisson noise, which models the corruption due to background photons, and (2) Gaussian noise, which represents the independent and identically distributed readout error for every pixel. SNR, which is commonly defined as the ratio of mean signal strength to standard deviation [21], has been documented as 24.6 for a previous single-shot PSP study on a hemispherical dome [47]. This corresponds to 4% noise (standard deviation = 0.04), which was added as random perturbations $\left({\left\|e\right\|}_{2}/{\left\|G_{2\mathrm{blurred}}\right\|}_{2}\right)$ to the blurred images following Hansen et al. [43]. To simulate the discretization error from the real world to the image plane, the images were constructed and blurred at a resolution of 3000 × 4000, and then downsampled to 600 × 800.

**Figure 5:**
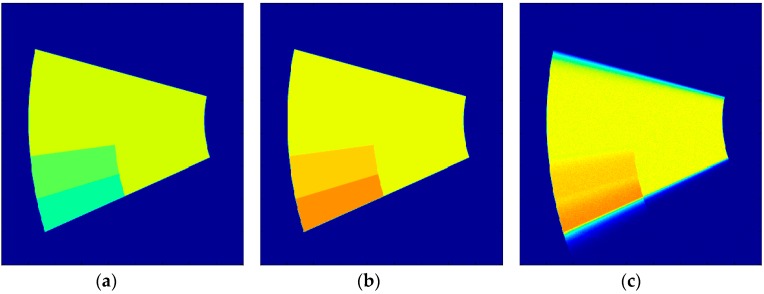
(**a**) Sharp Gate 1 image (3000 × 4000), (**b**) Sharp Gate 2 image (3000 × 4000), and (**c**) Blurred Gate 2 image for the rotating disk at 269 Hz downscaled (600 × 800) and with 4% noise added. Colors represent the intensity captured by the Gate 2 image. Rotation is counterclockwise; images are in Cartesian coordinates.

Samples of the forward-modeled sharp and blurred images used in this work are shown in Figure 5. For generating these, an image of a wedge-shaped region of PSP on a spinning disk is represented by pixels that have a value of 1 inside the paint, and 0 otherwise. This was used to represent the total luminescence intensity that each point on a uniform PSP disk emits after a single shot of pulsed laser. Depending on the pressure experienced, the lifetime of emission at each point varies and gets split into the two gates of the single-shot method, as described earlier. Three regions of pressures were selected in this elementary image—70 kPa (outer part of the leading edge), 90 kPa (the middle patch), and 110 kPa (remainder of the disk)—which produce the sharp Gate 1 and Gate 2 images, as shown in Figure 5a,b, respectively. It can be observed that depending on the pressure, the intensity of the regions is different, as captured in Gate 1 and Gate 2 images. To simulate anticlockwise rotation at 269 Hz, the three separate patches of the sharp Gate 2 image were then blurred separately as described earlier, and then superposed to form the blurred Gate 2 image. This high-definition blurred Gate 2 image (3000 × 4000) was then downsampled (600 × 800) to model the discretization error, and 4% noise was added to obtain the test image, as shown in Figure 5c.

### 4.2. Experimental Image

It has been pointed out by Levin et al. [34] that several classic papers on blind deconvolution do not work with real-world images. Hence, it is important to check the algorithm that has been developed in this work on images generated by actual PSP experimentation. In previous work, Gregory et al. [8] used an experimental image from a rotating disk setup to test the invariant deblurring scheme developed there; this same image experimental image will be used in the present paper to assess the spatially variant iterative scheme developed here.

The setup consisted of a spinning disk (radius of 101.6 mm), a segment of which was painted with PSP and imaged using a camera mounted above the disk in the laboratory frame. Since the radius of rotation was much smaller in comparison to a large scale rotor, a high rotational rate (134 Hz) was used to produce a comparable blur. In order to induce a sharp-edged gradient in local oxygen concentration (thus, emitted intensity), the setup had a provision for the tangential injection of a nitrogen jet across the disk surface. Thus, the setup allowed for the evaluation of a deblurring algorithm when applied to images with non-smoothly varying PSFs. The previous study [8] exposed the limitation of an assumed spatially invariant PSF for the restoration of such an image with sharp pressure gradients. The same blurred image has been used in this work to evaluate the iterative scheme developed here. For details about the experimental setup, the reader is referred to Gregory et al. [8]. 

**Figure 6:**
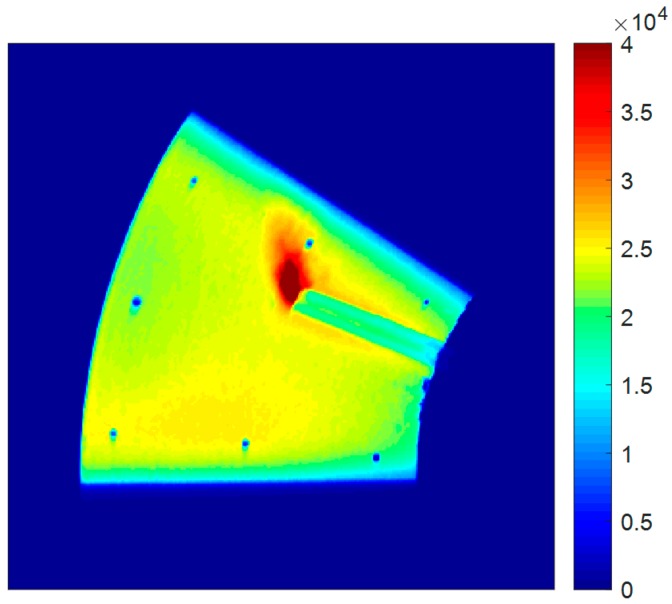
Blurred image of nitrogen jet grazing on rotating disk. Rotation is clockwise; image is in Cartesian coordinates. Colors represent intensity captured by Gate 2 of the 14-bit camera (adapted from Gregory et al. [8]).

Figure 6 shows the blurred Gate 2 experimental image with colors representing intensity captured by the 14-bit camera. It can be observed that the presence of PSP on the disk enabled the visualization of the colorless nitrogen jet, and the finite exposure time of the camera produced the blurring and smoothing of the jet profile. With respect to the PSP, the presence of the nitrogen jet has the same influence as a sharp decrease in pressure: it causes a decrease in the partial pressure of oxygen, and hence less oxygen-quenching. This leads to a longer lifetime of decay, giving a higher split of intensity in the second exposure. Even though the nitrogen jet follows a straight path upon exiting the rotating nozzle (when viewed in the inertial frame), the indicated jet trajectory is curved away from the direction of motion, since PSP visualizes the streaklines of the rotating jet. For computing the SNR, the technique proposed by Fang et al. [47] was used, in which a small patch (10 × 10 pixels) in a constant-intensity region was identified for ratioing the mean signal strength over standard deviation. The SNR in the recorded images was found to be as high as 90. Such a high SNR was one of the reasons (along with the low condition number of the blur kernel) that Weiner deconvolution, even without accounting for noise, produced reasonable reconstructions in Gregory et al. [8]. To study such large variations in SNR, two forward-modeled images, one with a SNR as low as 25 and another totally noise-free, have been used along with this experimental image (SNR of 90).

## 5. Results and Discussion

**Figure 7:**
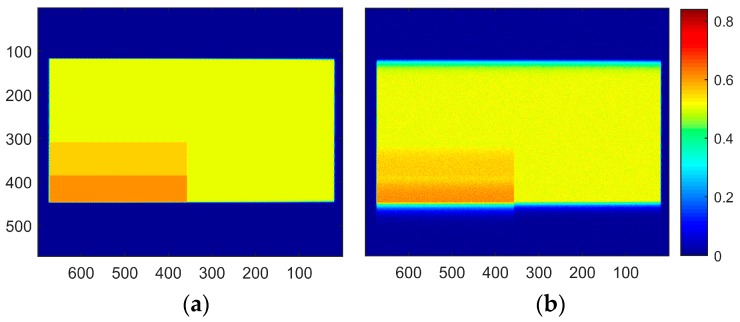
(**a**) Sharp image that is to be recovered in polar coordinates, and (**b**) blurred Gate 2 image (as in Figure 5c) in polar coordinates. Colors represent the intensity captured in the Gate 2 image. The image x-axis is pixels in the *r*-direction, and the y-axis is pixels in the *θ*-direction. Rotation is downward; images are in polar coordinates.

All of the figures (numerical and experimental) in this work have blades that were rotated in the counterclockwise direction. The first test case was used to simulate the application of the blind deconvolution scheme to experiments conducted at a low SNR, and understand the effect of regularization on the scheme. The elementary disk-shape forward-modeled image generated in Figure 5 was used for this purpose. The disk experiences three separate pressures with sharp changes in between them (Figure 5b), the precise locations of which cannot be ascertained in the downscaled, blurred, and noisy image (Figure 5c). A part of the blurred image that safely captures all of the luminescent decay is then transformed to polar coordinates with a radial resolution of 0.5 per pixel and an angular resolution of 0.1 per pixel to obtain a one-dimensional column-wise downward blurring. Figure 7 shows both the sharp Gate 2 image (a) and the blurred image (b) in polar coordinates.

**Figure 8:**
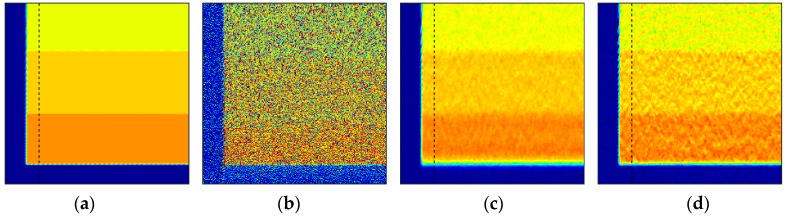
(**a**) Sharp image to be recovered, (**b**) result obtained using assumed *P* deconvolution without regularization, (**c**) polar restored image after nine iterations with five iterations of steepest descent, and (**d**) polar restored image after nine iterations with 15 iterations of steepest descent. Colors represent intensity. Dashed black line represents *r/R* = 0.95 used to plot profiles in Figure 9 and Figure 10. Axes are the same as in Figure 7. Rotation is downward; images are in polar coordinates.

The degraded image was restored using first the invariant-assumed deblurring [8] with a pressure of one atmosphere and without any regularization; then, it used the blind iterative scheme in conjunction with steepest descent implementation of Richardson method as described by Berisha and Nagy [25]. The results in polar coordinates are shown in Figure 8; Figure 8b is the unregularized result based on the spatially invariant PSF. Figure 8c,d show the results after nine iterations of the blind scheme with either five or 15 iterations of iterative regularization over each iteration, respectively.

**Figure 9:**
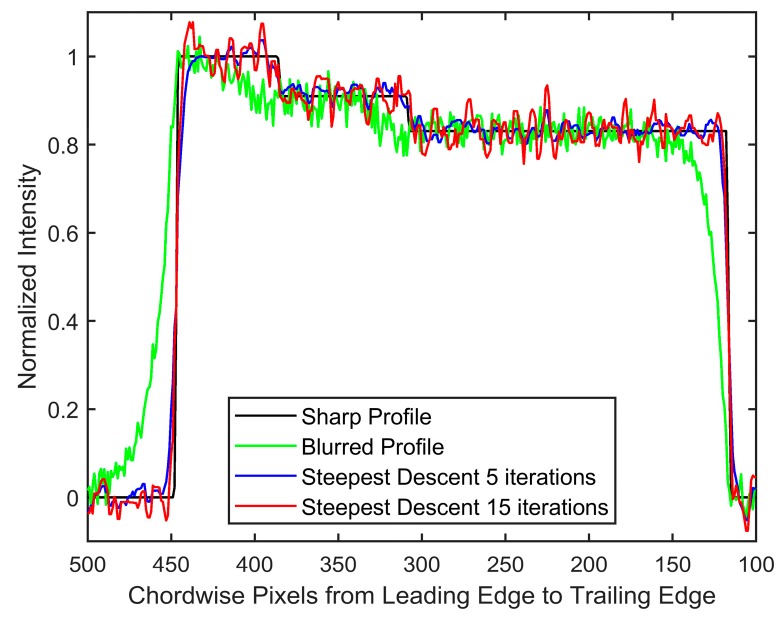
Effect of regularization iterations on restoration after nine iterations at *r* = 0.95. The x-axis represents pixels along the chord in polar coordinates. Rotation is toward increasing pixel values.

The importance of regularization is apparent, as noise amplification renders the spatially invariant deblurred image (Figure 8b) unintelligible. On the other hand, the blind scheme in conjunction with regularization tools restores the intensities to great accuracy while preventing the noise amplification (Figure 8c,d). Since the number of iterations acts as a regularization parameter in iterative methods, it can be observed that Figure 8d preserves high frequency information such as edge locations better, albeit with elevated noise. Figure 8c is much smoother, but the deblurring has also smoothed out the sharp intensity changes. Figure 9 shows a plot of intensity values at *r*/*R* = 0.95 (location identified by the dashed black line in Figure 8).

**Figure 10:**
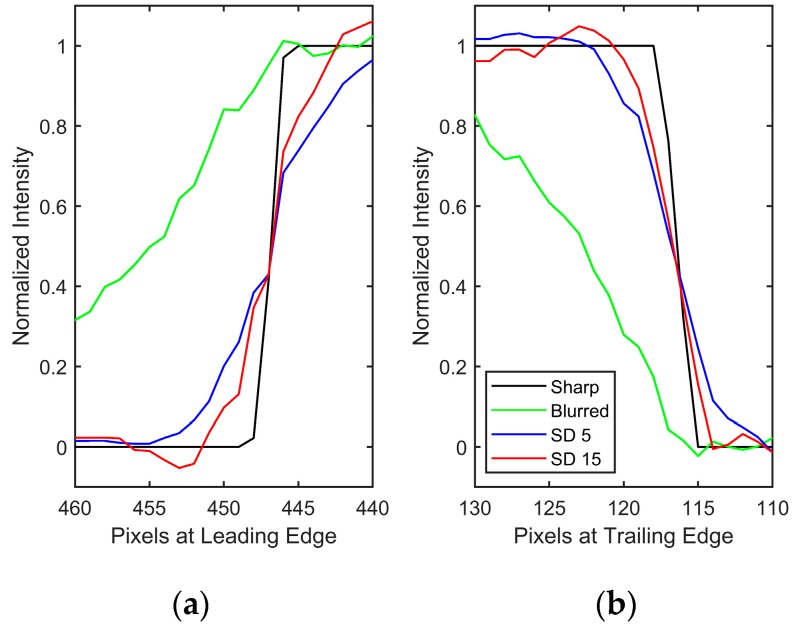
Comparison at the interface of 90 kPa and 70 kPa at *r*/*R*=0.95 at the (**a**) leading edge and (**b**) trailing edge. Rotation is toward increasing pixel values.

A zoomed-in version at the leading and trailing edges is shown in Figure 10 to show this effect of iteration count. High-spatial resolution reconstructions with high-frequency information are possible, as long as elevated noise levels can be tolerated (such as in high SNR experiments). The invariant case was excluded from this comparison due to the large amount of noise in the ‘restored’ image, but is considered in the next noise-free test case. Another feature to be noted is the effect of 2-norm-based regularization (Equation (6)) on the leading edge of the image. It has been known that 2-norm-based methods do not allow sharp gradients and produce smoother results [20]. Since important flow physics often have a first-order effect on the surface pressures near the leading edge of an airfoil, it is critical that a form of regularization that does not smooth out the intensity values be used to acquire the pressure information there. The total variation smoothing norm, which is based on 1-norm of the image gradients [48,49] is less harsh on gradients, and may be used to preserve the leading edge information in future work.

**Figure 11:**
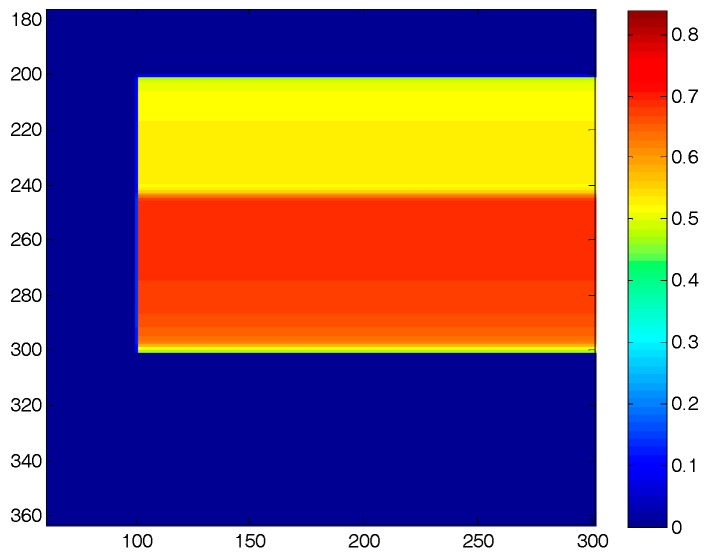
Sharp shock profile on a simulated propeller blade (to be recovered). Colors represent the intensity captured in the Gate 2 image, and axes represent pixels of the image in Cartesian coordinates.

A second test case was constructed using a pressure profile that is more representative of an actual aerodynamic test. The numerically generated image considered the case when a shock wave is present on a rotating blade, e.g., on an advancing blade in a high-speed wind tunnel. Figure 11 shows the outer edge (20% of the span is visible) of the sharp Gate 2 image of the propeller blade, where the blade is rotating in the counterclockwise direction. This intensity profile (which is inversely proportional to the pressure profile) is much smoother than that considered in the previous case (which only had two sharp discontinuities). Since resolution in noisy images depends on the regularization method employed, no noise was added in this test case in order to observe the intrinsic resolution of the blind deconvolution scheme.

**Figure 12:**
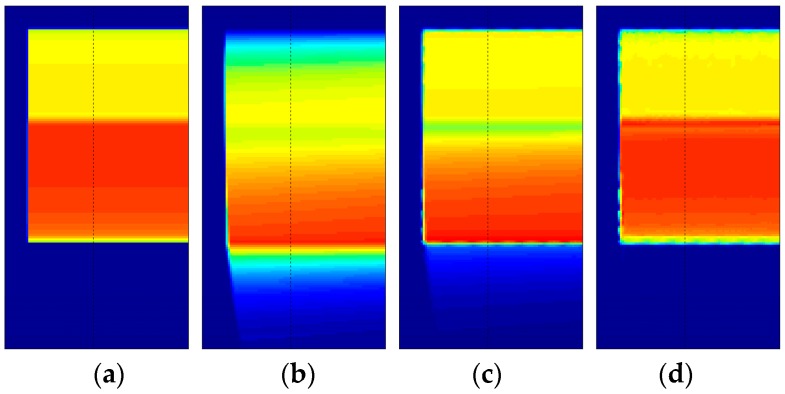
(**a**) Sharp intensity to be recovered, (**b**) blurred Gate 2 image obtained for blade rotation frequency of 269 Hz, (**c**) deblurred result as obtained from an invariant deblur with an assumed pressure of one atmosphere, and (**d**) recovered image after 13 iterations. Colors represent intensity. The dashed black line identifies the location (*r/R* = 0.95) used to plot profiles in Figure 13 and Figure 14. Axes are the same as in Figure 11. Rotation is counterclockwise; images are in Cartesian coordinates.

**Figure 13:**
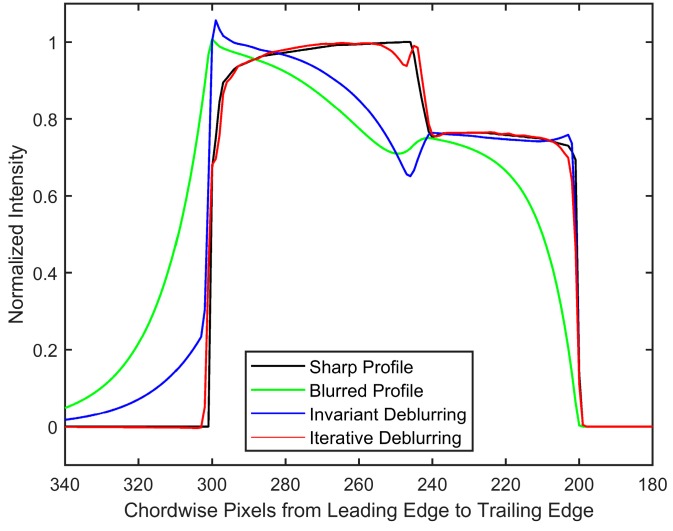
Comparison of applied and restored intensity values at *r*/*R* = 0.95. The iterative deblurring result is after 13 iterations. Rotation is toward increasing pixel values.

**Figure 14:**
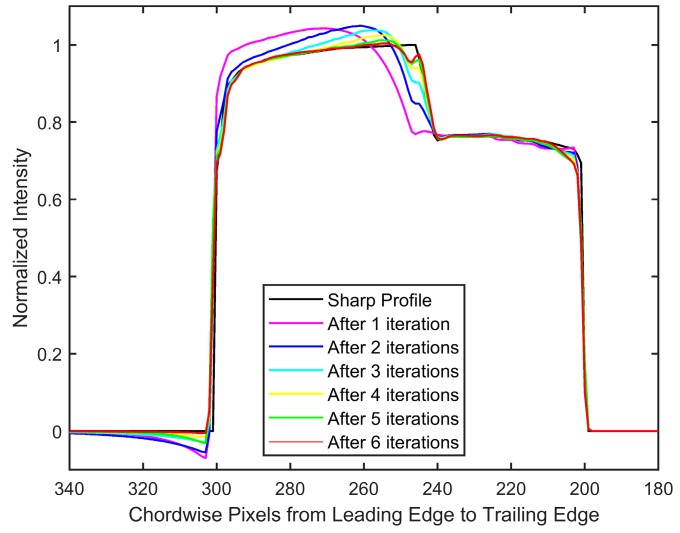
Restored intensity values after different iterations at *r*/*R* = 0.95. Rotation is toward increasing pixel values.

The polar lattice had a resolution of 0.5/pixel and 0.1/pixel in the radial and azimuthal directions, respectively. Due to the absence of noise, both invariant-supposed and spatially varying blind deconvolutions were simply carried out through the backslash operator in MATLAB. The results are shown in Figure 12. It can be observed that the assumed pressure of one atmosphere, as shown in Figure 12 c, is unable to restore the location of the shock, and brings to attention the inability of the invariant deblurring method when applied to PSP images with strong variations in pressure. On the other hand, the iterative method shows both qualitative and quantitative similarity to the initial intensity profile as not only the location, but also the intensity values are restored. This is more prominently seen in the intensity plot at *r*/*R* = 0.95 (Figure 13). The quality of restoration is very fine, as all of the pressure differentials are restored to correct locations, although a few differences in intensity values can be observed (particularly near the shock front). The intensity values after the first six iterations are shown in Figure 14, which shows that the convergence to a sharp image is expeditious.

**Figure 15:**
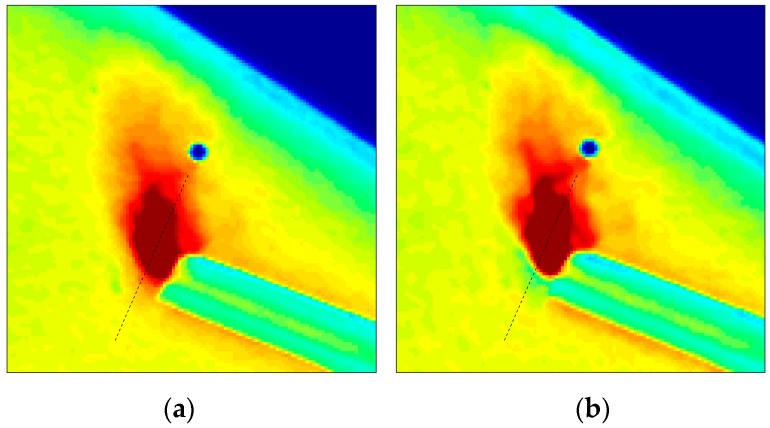
Comparison of (**a**) invariant and (**b**) iterative restorations for the rotating disk. The dashed black line represents the location that was used to plot the profile in Figure 16. Color represents the intensity captured in the Gate 2 image. Rotation is counterclockwise; images are in Cartesian coordinates.

The rotating disk image with a grazing nitrogen jet serves as a real-world problem for assessing the robustness and quality of the deblurring schemes. Since a rotating disk has a theoretically uniform pressure of one atmosphere, invariant deblurring is able to produce reasonable results over much of the surface of the disk. It can be seen in Figure 15a that the registration holes are restored back to their circular shape. However, the presence of the nitrogen jet on the disk changes the pressure values, which stipulates that appropriate PSFs should be used to reconstruct the correct jet profile. The blind iterative scheme finds these pressure values and employs the corresponding PSFs to restore the sharpness of the jet profile that was blurred during the second exposure. The results, which are shown here after 16 iterations (Figure 15b), can be used to locate the exact position of the pressure change. The resolution of the polar lattice that is used to transform the blurred image (Figure 6) was 0.5/pixel and 0.1/pixel in the radial and azimuthal directions, respectively. Since a high SNR of 90 was found in this experiment, 50 iterations of the Richardson method over every iteration of the blind scheme has been used to suppress the noise in the reconstructions.

**Figure 16:**
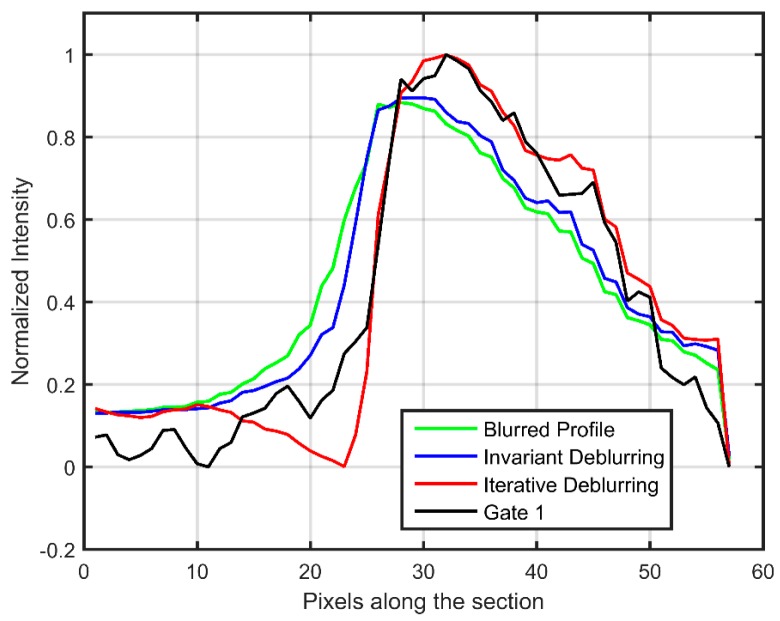
Intensity values for pixels on a section passing through the jet perpendicular to the sense of rotation. Rotation is toward decreasing pixel values.

The intensity values of pixels on a section passing through the jet tangential to the sense of rotation are plotted in Figure 16. For comparison, the intensity profile from Gate 1 is also plotted, since it undergoes negligible blurring due to the short exposure of 5 µs, and when normalized serves as the true intensity profile that needs to be recovered. Since the accumulated intensity is different in the two gates, Gate 1 has been normalized by itself, whereas the blurred and deblurred Gate 2 profiles have been normalized by the iteratively deblurred profile. The plot demonstrates that due to the spatial variation in pressure, invariant deblurring using a PSF based on the assumption of uniform pressure of one atmosphere is unable to restore the intensity values to their original location. Correspondingly, the location of the sharp gradient is erroneous, and is close to that indicated by the blurred image. On the other hand, iterative deblurring is able to identify the exact location of the sharp pressure change, and the profile matches closely with the unblurred Gate 1 profile.

Another issue with invariant deblurring can be observed; the use of a smaller PSF for one atmosphere pressure is unable to restore the longer decay that is associated with smaller pressure in the nitrogen jet, and thus exhibits about 10% smaller intensity values. This could lead to significant errors in indicated pressure if iterative blurring is not used. A sharp excursion at the beginning and the end of the profile is observed, which was attributed to the Gibbs ringing phenomena in Gregory et al. [8] and is commonly observed in image deblurring studies with sharp gradients. The rotating tube through which the jet is emanating undergoes a more complex blur as it collects and emits luminescent intensity from various points on the nearby surface. Hence, the PSFs at those locations are not accurate. However, since this feature will not be encountered in an actual PSP experimentation, it is here deblurred by the same procedure.

## 6. Conclusions

An iterative blind deconvolution method with a pixel-to-pixel spatially varying blur kernel is developed in the present work in order to restore the long second-exposure images of rotating surfaces in a single-shot method of PSP experimentation or other long-exposure PSP studies. The algorithm deblurs a single blurred Gate 2 image using the same three complementary images—wind-on Gate 1 and wind-off gate 1 and 2—that are needed in the single-shot method to obtain the pressure information. Since the blurred image contains pressure information, it is not subjected to any filtering in the deblurring scheme, and all of the processing is done on the restored image to generate an improved blur kernel. Paint characteristics, including pressure sensitivity and decay constants, are a necessary input to this scheme.

The convergence of this scheme to the sharp Gate 2 image is ensured by the monotonic behavior of lifetime decay with respect to pressure. Since only a part of the PSP image captures the blurred rotating surface, a highly resolved surface pressure map is obtained by processing this part of the image in upsampled polar coordinates. This resolved surface pressure map enables the creation of sub-pixel PSFs for the refined restoration of the blurred image. The restored image is then transformed to the same Cartesian coordinates by padding zero-intensity pixels for representing the background, which was not transformed to polar coordinates. The zero boundary condition is used to create the blur kernel, which results in a block triangular sparse matrix with triangular blocks. PSFs are positioned at the main diagonal of this lower triangular matrix.

A kernel-free forward model was used to simulate the splitting of intensity between the two exposures, as well as the blurring process during the second exposure for producing degraded images with known pressure and intensity values. Noise was added in the images to account for the errors and variations in the SNR in the PSP experimentation. Since image deblurring is an ill-posed problem and SVD-based filtering is infeasible for large data sizes, the Iterative Regularization Tools of Nagy and coworkers [25,26] were used over every iteration of the blind scheme to suppress noise amplification. Test images corresponding to a low SNR of 25 were used to show the effect of regularization iteration count on the reconstructions. As expected, a lower count leads to a smoother image, but cannot be used to accurately locate a sharp pressure change, whereas a higher count preserves high-frequency information, but with higher noise. The inherent resolution of the blind scheme was evaluated on a noiseless shock profile, which revealed the high accuracy of the reconstructions and improvement over the previously used invariant restoration in PSP images. A real-world problem was then used to show how this deblurring method can help find the accurate location of the pressure differential and their values.

Although this specific algorithm is limited to PSP and TSP data images, it presents an effective solution to the deblurring requirement in single-shot method, which could not be met by any other restoration algorithm. The deblurring work done in this paper may lead to the development of longer lifetime PSPs with confidence for improved sensitivity, which will help resolve flow physics not being captured by current PSP formulations.
